# Suppression on plant-parasitic nematodes using a soil fumigation strategy based on ammonium bicarbonate and its effects on the nematode community

**DOI:** 10.1038/srep17597

**Published:** 2015-12-01

**Authors:** Lanxi Su, Yunze Ruan, Xiujuan Yang, Kang Wang, Rong Li, Qirong Shen

**Affiliations:** 1 Jiangsu Key Lab for Solid Organic Waste Utilization, National Engineering Research Center for Organic-based Fertilizers, Jiangsu Collaborative Innovation Center for Solid Organic Waste Resource Utilization, Nanjing Agricultural University, 210095, Nanjing, China; 2 Hainan key Laboratory for Sustainable Utilization of Tropical Bio-resources, College of Agriculture, Hainan University, 570228, Haikou, China

## Abstract

Banana production is severely hindered by plant-parasitic nematodes in acidic, sandy soil. This study investigated the possibility of applying a novel fumigation agent based on ammonium bicarbonate as a strategy for controlling plant-parasitic nematodes under sealed conditions. Moreover, its effects on the nematode community in pot and field experiments were also measured using morphology and feeding-habit based classification and the PCR-DGGE method. Results showed that a mixture (LAB) of lime (L) and ammonium bicarbonate (AB) in suitable additive amounts (0.857 g kg^−1^ of L and 0.428 g kg^−1^ of AB) showed stronger nematicidal ability than did the use of AB alone or the use of ammonium hydroxide (AH) and calcium cyanamide (CC) with an equal nitrogen amount. The nematode community was altered by the different fumigants, and LAB showed an excellent plant-parasitic nematicidal ability, especially for *Meloidogyne* and *Rotylenchulus*, as revealed by morphology and feeding-habit based classification, and for *Meloidogyne*, as revealed by the PCR-DGGE method. Fungivores and omnivore-predators were more sensitive to the direct effects of the chemicals than bacterivores. This study explored a novel fumigation agent for controlling plant-parasitic nematodes based on LAB and provides a potential strategy to ensure the worldwide development of the banana industry.

Bananas are a staple food because of their high protein content and nutritional value, the main source of income in many developing countries^1^, and an important food security crop, providing a cheap and easily produced source of energy^2^. However, the production of bananas is hampered by many diseases and pests^3^, including plant-parasitic nematodes, which are widespread and among the most damaging pests of banana. Plant-parasitic nematodes cause root damage that not only leads to severe crop losses in commercial banana plantations for export but also seriously limits the production and viability of all banana types^4^,^5^. More than 151 nematode species of 51 genera have already been documented worldwide on banana^6^. All banana varieties are hosts of root-knot nematodes belonging to the *Meloidogyne* genus, which attack many economically important crops, cause deformation and stunting of the roots, and are more likely to be found in great numbers on banana roots in the tropics^7^. All bananas are also host to the *Pratylenchus, Tylenchus, Ditylenchus* and *Helicotylenchus* genera, which are also major nematode pests and affect banana production across the globe^8^. Thus it is both necessary and urgent to find a better way to ensure healthy banana crops and maintain worldwide development of the industry.

To fight banana root nematodes, cultural, biological, physical and chemical control methods are used^9^. However, control options are limited because in most systems of cultivation, bananas are grown as a permanent crop, with no available nematode-resistant varieties^10^, and no specific nematode control methods are yet practiced in traditional cultivations using nematicides^11^. Furthermore, biocontrol is still infeasible, and the effect of mulches on nematode populations is not clear^12^. What’s more, increasing public interest in protecting the environment and human health has prompted research focused on agronomic strategies that reduce the use of fungicides, fertilizers and herbicides^13^; thus, environmentally friendly alternative methods for managing plant-parasitic nematodes have to be developed.

Soil pretreatments, such as soil fumigation with methyl iodide, propargyl bromide, 1, 3-dichloropropene^14^, calcium cyanamide^15^ and methyl bromide, are very effective in suppressing nematode populations^16^. However, due to the destruction of soil ecosystems by many of these chemicals, the use of fumigant pesticides is restricted^17^,^18^; for example, calcium cyanamide is no longer available because of the risks it posses to environmental health^19^,^20^. Various physical methods, including steam disinfection, soil solarization and hot water injection, have also been employed with varying success for the control of nematodes as alternatives to soil fumigation with synthetic chemicals. However, many factors, including the soil type, climatic conditions and water content of the soil, can affect the effectiveness of physical treatments^21^. Thus, it is necessary to find a better soil pretreatment method.

Ammonia has been proved to be responsible for many effects on nematodes: some plant parasitic nematodes are very sensitive to low concentrations of this compound and die^22^,^23^,^24^. On the other hand, different attraction-repulsion effects produced by ammonia have been reported as a response of entomopathogenic nematodes for seeking a new host^25^,^26^,^27^. Upon reaction with soil moisture, calcium cyanamide breaks down initially to hydrated lime and hydrogen cyanamide, which then forms ammonia in sequence^28^, especially ammonia, being reported to reduce the number of nematodes on tomatoes when used as ammonium nutrition^29^. The use of bicarbonate salts was not expected to be harmful to humans and the environment^30^. Since then, among the ten ammonia-releasing compounds that have been tested, ammonium hydroxide, ammonium phosphate and ammonium bicarbonate have been observed to show the greatest nematicidal activity in pots, and ammonium hydroxide was found to be the most nematicidal of these compounds^23^. The enclosure of pots containing ammonia-releasing materials and treated soils in plastic bags reduced the concentration of the materials needed to kill *Meloidogyne javanica*^31^. However, no reports, to our knowledge, have described the effects of soil pretreated with different ammonia production substances as soil fumigants on the banana-parasitic nematode population and the whole nematode community structure, especially in acidic, sandy soils with continuous banana plantings over several years that are seriously infected with plant-parasitic nematodes that harm banana production.

The use of ammonia-releasing materials combined with lime as a soil fumigant to reduce the acidity or increase the alkalinity of the soil has led to interest in these substances as a nematicidal agent. In this study, several ammonia production materials were evaluated as soil fumigants as an environmentally friendly, safe and long-lasting mean of nematode prevention in acidic, sandy soil in southern China. The objectives of this study were to compare the effect of several ammonia production substances as soil agents suppressing nematodes, to identify the best nematicide disinfectant, to explore the optimum application mode of the disinfectant, and to investigate the effects of different applied disinfectants on the soil nematode community structure.

## Results

### Nematicidal effect of LAB

**Figure 1:**
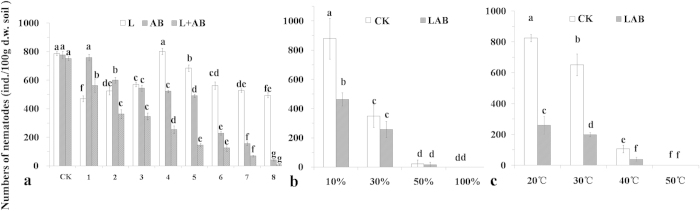
Effects of different concentrations of L, AB and LAB on the number of nematodes (a); effects of water content (b) and temperature (c) on the suppressed nematodes ability of soils treated with 0.857 g of L and 0.428 g of AB. The error line means standard deviation of the mean. Bars with different letters indicate significant differences of the different concentrations for each treatments (**a**) and among the treatments (**b**,**c**) as defined by Duncan’s test (P < 0.05).

The effects of different concentrations of L, AB and LAB on nematode numbers are shown in Fig. 1a. For all the results, the maximum population was found in the CK (pot soils with no additives), AB1 and L4. The total number of nematodes showed a continuous decrease in the AB and LAB treatments and an irregular fluctuation in the L treatment. The minimum value was observed after treatment with AB and LAB in the highest amount added, and less than 300 individuals per 100 g d.w. soil were identified when the amount added was more than 0.857 g of L and 0.428 g of AB. Thus, this concentration (LAB4) was selected for further study. Compared with the AB treatment, the LAB treatment improved the effects for reducing the nematodes numbers by 51.4%, 70.4%, 45.0%, 55.7% and 83.7% with the increase of additional concentrations, respectively.

For treatments with different soil moisture contents and the control, the total number of nematodes followed the same trend, gradually decreasing with increasing soil moisture content. Treatment with LAB significantly reduced the nematode number compared with the control when the soil moisture content was 10%, while no significant difference was observed when the moisture was higher than 30%, which is unsuitable for survival (Fig. 1b). For treatments with different temperatures compared with the control, the number of nematodes in the soil gradually decreased with increasing temperature values. The nematode number in the soil of the LAB treatment was significantly lower than that of the control when the temperature was less than 40 °C (Fig. 1c).

### Physicochemical properties of soil samples from LAB treatments at different concentrations

Compared with the previously published results for different nematicidal agents, the utilization of LAB more efficiently killed the nematodes, representing the most efficient strategy. Therefore, the physicochemical properties of soil samples treated with different concentrations of LAB and the control (CK) with no additives were determined. As shown in Table 1, the pH value overall continuously increased with increasing amounts of LAB and higher than that in CK; the NH_4_^+^-N content increased to 28.99 mg kg^−1^ in the 2 treatment (0.428 g of Lime and 0.214 g of ammonium bicarbonate), slowly decreased to 11.70 mg kg^−1^ when 2.568 g of lime and 1.284 g of ammonium bicarbonate were added, and then suddenly increased to the highest value of 136.23 mg kg^−1^ in the treatment with the highest addition amount. The NO_3_^−^-N content continuously increased with increasing addition of LAB when the amount added was less than 3.424 g of lime and 1.712 g of ammonium bicarbonate. After this, the NO_3_^−^-N content suddenly decreased to the lowest value of 29.64 mg kg^−1^. The total nitrogen content of the soil samples increased with increasing LAB, ranging from 0.36 mg kg^−1^ in the a treatment to 0.51 mg kg^−1^ when the highest amount was added, representing the accumulation of nitrogen in the soils with added LAB.

### Nematicidal effect of different ammonia production materials in Petri dishes

**Figure 2:**
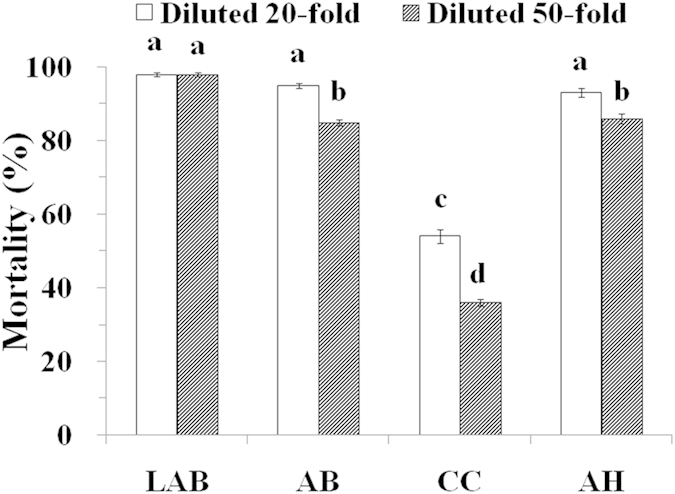
Effects of 20- and 50- fold dilutions of LAB (0.857 g of lime and 0.428 g of AB, d.w.), AB (0.428 g of AB, d.w.), CC (0.257 g of calcium cyanamide, d.w.) and AH (0.139 g of ammonium hydroxide, d.w.) on the corrected mortality of nematodes. The error line means standard deviation of the mean. Bars with different letters indicate significant differences among the treatments, as defined by Duncan’s test (P < 0.05).

The nematicidal effect of aqueous solutions of LAB, AB, CC and AH evaluated in a Petri dish experiment are shown in Fig. 2. Both diluents of LAB almost completely killed the nematodes. In the 20-fold diluent experiment, compared with the control (Petri dish with no additives) with mortality of 12%, the corrected mortality of the nematodes for LAB, AB, CC and AH treatments were 98%, 95%, 54% and 93%, respectively. In the 50-fold diluent experiment, the values were 98%, 85%, 36% and 86%, respectively. These Petri dish experiment results indicate that LAB is the most efficient nematicidal agent.

### Nematicidal effect and nematode community analysis after fumigation in the pot experiment by morphology

For the total number of nematodes after fumigation, as shown in Table 2, no significant difference was observed in the nematicidal effects between AB and AH treatments. Compared with these two treatments and L control, the application of LAB significantly reduced the nematode number, indicating that LAB is the most efficient soil pretreatment agent. To determine the effects of different ammonia production materials on the nematode distribution, soil samples from the pot experiment collected after fumigation were selected for nematode taxa analysis. As shown in Table 2, all trophic groups were significantly affected by fumigation. No significant difference in the number of bacterivores was observed among the three treatments and L control, while the values were significantly lower than that in CK. The LAB and AH treatments significantly decreased the fungivores number compared to the AB treatment and CK and decreased the omnivores-predators number compared with L and CK. Both LAB and AB killed more plant parasites than the AH treatment and CK.

A total of 17 taxa were counted and identified in the soil samples collected after fumigation (Table 2). Bacterivores, with 7 taxa, were the most diverse trophic group. No significant difference of the *Acrobeloides* number was observed in the AB, LAB, AH treatments and L control, and the values were significantly lower than that in the CK. The AB, LAB and AH treatments significantly decreased the *Chiloplacus* number compared to CK. Moreover, the LAB and AH treatments and L control significantly decreased the *Cephalobus* number compared with the CK and AB treatment, while LAB and CK showed no significant difference in *Mesorhabditis* number, which significantly increased in the L control and AH treatment. No significant difference in the number of *Prismatolaimus, Acrobeles* and *Monhystera* were observed among the treatments and controls. Four species of plant parasites were identified in the soil samples: *Rotylenchulus, Meloidogyne, Pratylenchus* and *Tylenchus*. The LAB and AH treatments showed the best fumigation effect for *Rotylenchulus* compared to CK, L and AB treatment. The numbers of *Meloidogyne* in the AB and LAB treatments were significantly lower than in other treatments and the two controls. Significantly lower numbers of *Tylenchus* were observed in the L, AB and LAB treatments, followed by the AH treatment, while the values were highest in the CK. All treatments and controls showed no significant difference in *Pratylenchus* number. The number of *Tylencholaimus, Aphelenchus* and *Aphelenchoides*, belonging to fungivores, and *Mylonchulus, Oxydirus* and *Aporcelaimus*, belonging to omnivores-predators, differed for all treatments and controls.

### Nematicidal effect and nematode faunal analysis at the end of the pot experiment by morphology

**Figure 3:**
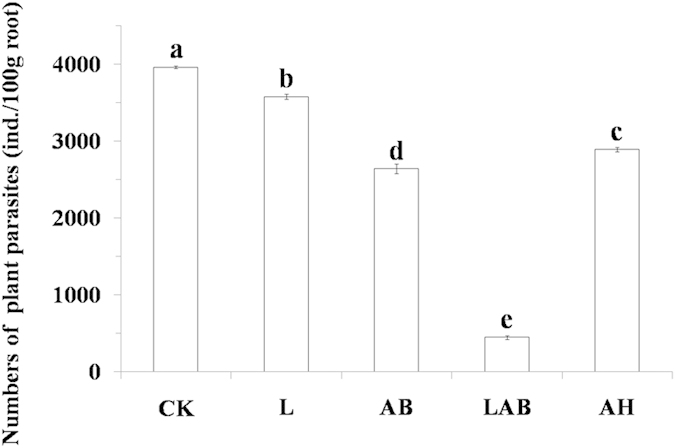
The total number of plant parasitic nematodes (ind./100 g root) in roots in CK and L controls and AB, LAB and AH treatments after harvest during the pot experiment. The error line means standard deviation of the mean. Bars with different letters indicate significant differences among the four treatments, as defined by Duncan’s test (P < 0.05).

At the end of the pot experiment, significant differences in the numbers of plant parasites and total nematodes were found among the three treatments and two controls for both the banana roots and the soil, and the lowest values were observed in the LAB treatment, followed by the AB treatment, AH treatment, L control and CK (pot soils with no additives) for roots (Fig. 3) and by the AB treatment, L control, AH treatment and CK for soil (Table 2).

For the tropic groups in the soil, LAB treatment showed the highest number of bacterivores. No significant difference in the fungivores number was observed among the treatments, and the values were significantly lower than those in the CK. Significant difference in the numbers of plant parasites was observed among the three treatments and the two controls. The LAB treatment showed the lowest number, followed by the AB treatment, L control, AH treatment and CK, indicating that LAB is the best fumigation agent. The highest number of omnivores-predators was observed in the L, LAB treatments and CK, followed by the AB and AH treatment (Table 2).

A total of 15 taxa were counted and identified in the soil samples collected at the end of the pot experiment (Table 2). The LAB treatment showed the highest number of all 6 taxa belonging to bacterivores: *Mesorhabditis, Acrobeloides, Cephalobus, Prismatolaimus, Chiloplacus* and *Acrobeles*, with the first three significantly higher in number compared with the other treatments and with the controls. No significant differences in the number of *Prismatolaimus* and *Acrobeles* were observed among the treatments and controls. No significant difference in the number of *Tylencholaimus* belonging to the fungivores group was observed among the treatments and the L control, and the value was significantly lower than that in CK. Four species of plant parasites were identified in the soil samples: *Rotylenchulus, Meloidogyne, Pratylenchus* and *Ditylenchus*; in the LAB treatment, the *Rotylenchulus* was present in the lowest number among all the treatments. Compared to the other treatments and controls, the LAB and AB treatments also showed a fumigation effect toward *Meloidogyne*. The lowest number of *Pratylenchus* was observed in the L control and the all treatments, while the values were highest in the CK. Among the omnivores-predators, the number of *Oxydirus* was not significantly different among the samples, and the LAB treatment showed the highest value of *Microdorylaimus*. The CK control showed the highest value for *Mylonchulus*, while the highest number of *Achromadora* was observed in the L control.

### Nematode community analysis after fumigation in the pot experiment using PCR-DGGE

**Figure 4:**
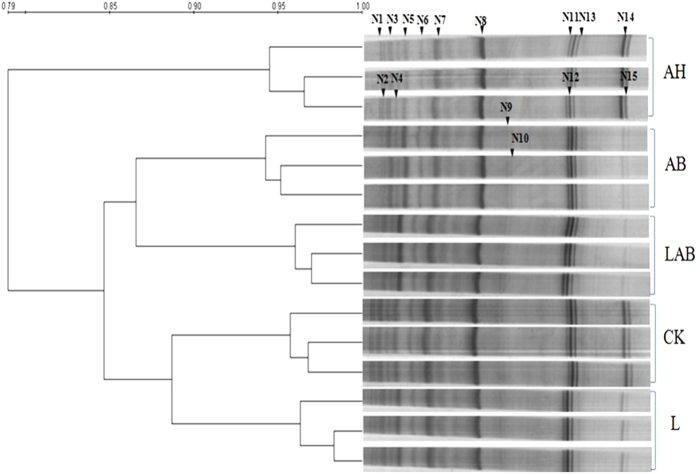
Nematode community associated with CK and L controls, AB, LAB and AH treatments after fumigation of the pot experiment, as determined by PCR-DGGE. The cluster diagram of the PCR-DGGE fingerprints from the different soil samples is based on the 18S rRNA nematode gene. Profiles were analyzed using the Dice coefficient and a UPGMA algorithm. Bands marked were excised, cloned and sequenced.

The DGGE fingerprint analyses of the soil samples after fumigation are shown in Fig. 4. Based on a UPGMA method analysis, the DGGE lanes grouped into 3 clusters were distinctive. The three-replicate cluster for the soil treated by LAB was similar to that from the AB treatment and different from the other clusters (Fig. 4).

The strengthened bands in the DGGE gels were excised for sequencing, and 15 bands were successfully sequenced and analyzed by a BLAST search. The highest identities based on comparison to known and putative species in the NCBI database are shown in Table 3, and all the identified bands belonged to plant-parasites and bacterial-feeders. For the plant-parasites, the weakened bands (N14 and N15) detected in the LAB and AB samples were identified as *Meloidogyne javanica* and *Meloidogyne incognita.* The N5 and N11 bands observed in all the samples were identified as *Tylenchus arcuatus* isolate wb8 and *Helicotylenchus dihystera* and were weakly strengthened in the LAB and AB treatments. No obvious differences in intensity were observed for bands N7, N8 or N12, identified as *Ditylenchus brevicauda, Rotylenchulus reniformis* and *Rotylenchulus reniformis*, respectively. For the bacterial-feeders, no obvious differences in the intensities of the bands were detected, except for in bands 1 and 2, identified as *Prismatolaimus intermedius* and *Cephalobus cubaensis*, respectively, which showed decreased intensities in the LAB and AB treatments.

### Nematode community analysis at the end of the pot experiment using PCR-DGGE

**Figure 5:**
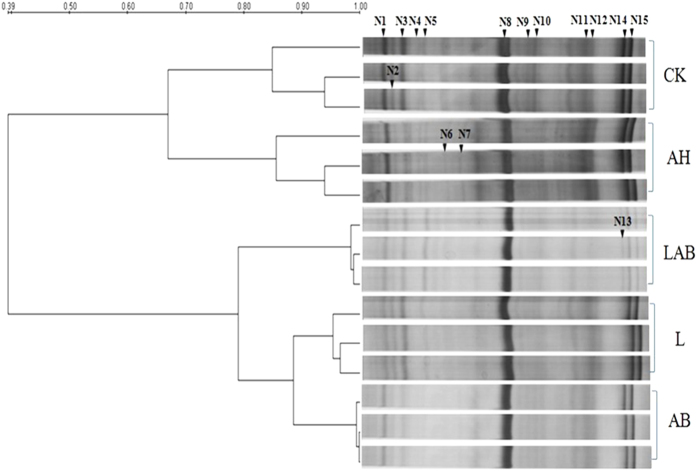
Nematode community associated with the CK and L control and the AB, LAB and AH treatments at the end of the pot experiment as determined by PCR-DGGE. The cluster diagram of the PCR-DGGE fingerprints from different soil samples is based on the 18S rRNA nematode gene. Profiles were analyzed using the Dice coefficient and a UPGMA algorithm. The bands marked were excised, cloned and sequenced.

The DGGE fingerprint analyses of the soil samples collected at the end of the pot experiment are shown in Fig. 5. Based on a UPGMA method analysis, the DGGE lanes grouped into 2 clusters were distinctive. The three-replicate cluster for the soil amended with LAB was distinct from that of the other samples.

The strengthened bands in the DGGE gels were excised for sequencing, and 15 bands were successfully sequenced and analyzed by a BLAST search (Table 4). For the plant-parasites, the weakened bands (N3, N4, N6, N7, N11, N12, N14 and N15) detected in the LAB and AB samples. Band 14 and 15 were identified as *Meloidogyne javanica* and *Meloidogyne incognita*; N8, identified as *Rotylenchulus reniformis*, showed no difference among all the samples observed. For the bacterial-feeders, the majority of the bands in the treatments of AB and LAB and in the L control were weaker than in the AH treatment and in CK.

## Discussion

This study, to our knowledge, marks the first investigation of a novel fumigation agent to be applied to soil as a strategy for controlling plant-parasitic nematodes in acidic, sandy soil which was monocultured banana for more than ten years. Because ammonium bicarbonate under alkaline conditions is more readily volatilized into ammonia^32^ and the nematicidal activity of ammonia and ammonia-releasing organic amendments is generally stronger in alkaline soils^33^. Both ammonium bicarbonate alone and ammonium bicarbonate with lime (two-fold dose relative to ammonium bicarbonate) were investigated as fumigation agents in nematode-affected soil in Hainan province, China, of which the pH value generally reported is less than 7^13^. Different concentrations of ammonium bicarbonate alone and ammonium bicarbonate with lime were tested, revealing that higher additive amounts produced enhanced ability for suppressing nematodes population. The result was supported by earlier findings that ammonium bicarbonate was reported to suppress *Belonolaimus longicaudatus*, one kind of plant-parasitic nematode, in the field^34^. In addition, the mixture of ammonium bicarbonate and lime possessed significantly higher suppressing effects compared to the same dose of ammonium bicarbonate alone, most likely due to the higher pH value generated by the addition of lime^32^. The soil pH is consequently the crucial driving factor in the production of ammonia^32^. In acid soils, ammonium-nitrogen (NH_4_^+^-N) predominates, while at higher pH, NH_3_ would be more prevalent^35^. When the concentrations of the mixture were more than that in LAB4, pH was greater than 7, also showing a better nematicidal ability. The application of excess ammonia-releasing and ammonium compounds is not only phytotoxic and costly; it may also contaminate ground water through the leaching of nitrate, which is produced from ammonia by the activity of soil microorganisms^23^. Our results showed that the nematode number was less than 300 individuals per 100 g d.w. soil when the amount of the mixture added was more than 0.857 g kg^−1^ of L and 0.428 g kg^−1^ of AB, indicating a suitable dose to be selected for further study. In this experiment, an increasing total nitrogen content with increasing additive amounts were also observed, while the NH4^+^-N content increase did not correspond with the additive amount due to the soil ammoxidation ability^23^. In addition, LAB was further confirmed to be the most effective fumigation agent when compared to the suppressing effect of AB, calcium cyanamide which was previously reported to be an effective fumigation agent^36^, and ammonium hydroxide which was also observed to be the most nematicidal compound compared with ten ammonia-releasing compounds through pot experiments^23^. The superior effect of LAB compared with ammonium hydroxide may be because the latter one is not suitable where the soil texture makes sealing of the tine furrow difficult; for example, on very heavy or sandy soils^37^. The density of nematodes was reduced in wet sites. The soil under the low oxygen condition produced the anaerobic metabolism product which can suppress the number of nematodes^38^,^39^. In our study, the mortality is so high when the soil moisture content was greater than 50%.

During one season of pot experiments, the nematode community structure was investigated by morphology and feeding-habit based classification because nematodes have high species diversity and belong to different feeding groups, including bacterivores, fungivores, plant parasites and omnivores-predators^40^, and are ubiquitous members of the soil faunal community in terrestrial ecosystems^41^. It is particularly important and significant to investigate the effects of fumigation on the plant-parasitic nematode population and community structure. Plant-parasitic nematodes which are among the most difficult crop pests to control may reduce crop yield through direct cell destruction and the vectoring of viruses or indirectly by facilitating the invasion of fungi and bacteria through their feeding and movements through roots^42^. Our results showed that, LAB was an excellent fumigation agent and showed a better suppression ability for *Meloidogyne* than AH treatment and the two controls after fumigation and harvest, and for *Rotylenchulus* than all treatments and the two controls after harvest. No significant difference in the values of *Meloidogyne* number in LAB and AB treatments was observed after fumigation and harvest, nevertheless, the values in the LAB treatment were both lower. *Meloidogyne* is the major nematode pest affecting banana (*Musa* spp.) production as well as a wide range of other agricultural crops across the globe^11^,^43^. *Rotylenchulus reniformis* can suppress crop yield of 60–74%^44^. Similar observations that soil treated with different fumigants, such as methyl iodide, 1, 3-dichloropropene, or propargyl bromide, successfully suppress plant-parasitic nematodes, especially *Mesocriconema* and *Meloidogyne*, have previously been reported^16^. However, many of these alternative soil fumigants are devastating to soil ecosystems^21^. The alternative fumigant described in the present study, which has previously been used as a general inexpensive fertilizer, may be widely pursued. In addition, our nematode community structure results also indicated that the abundance of fungivores and omnivores-predators was strongly reduced by all fumigants, most likely due to direct fumigant toxicity and alterations to the fungal decomposition channel^45^, while the abundance of bacterivores did not differ extensively among the treatments. In agreement with our results, previous studies have also shown that fungivores were more sensitive to the direct effects of chemicals than were bacterial-feeders^45^.

However, morphology and feeding habit-based classification exhibits serious limitations in terms of taxonomic identification and low sample throughput^46^. Only experts with the knowledge and skills to identify nematodes can conduct such analyses on a regular basis, which poses a major obstacle in the analysis of nematode communities^47^. Thus, PCR and sequence-dependent molecular biological methods, including denaturing gradient gel electrophoresis (DGGE), were applied to further study the nematode communities. The nematode primers M18F and M18R from Bhadury and Austen^48^ were used to fingerprint nematode diversity. After fumigation and at the end of the pot experiment, as revealed by PCR-DGGE, LAB application increased the nematode community structure (i.e., more bands); Based on an UPGMA method analysis of the DGGE lanes, the LAB treatments clustering with the AB treatment were distinctive to AH, CK and the L control after fumigation and then AH and CK at the end of the pot experiment, indicating that LAB application significantly altered the nematode community.

Most of the bands excised from the gels demonstrated high sequence similarity with nematodes, showing that the modified primer and the DGGE target resolved the nematode 18S rDNA signal in these soils^49^. The nematode species known to cause the most serious damage to bananas are those belonging to the migratory endoparasites, including the sedentary parasite *Meloidogyne* spp.^10^. In the present study, the results of morphology and feeding habit-based classification and the DGGE both revealed that the LAB treatment resulted in effectively suppressing ability for plant-parasitic population. Although the presence of bands representing different nematode taxa were relatively weaker than those observed by morphology and feeding-habit based classification, elucidating the factors that determine the abundance and composition of soil nematode communities is still a promising approach when investigating and understanding the effects of different fumigants in this study.

According to reports, ammonia is the source of more than 95% of the chemical nitrogen fertilizer currently produced in the world, and may be used directly as a fertilizer or converted to ammonium salts, nitrates, or urea^50^. It is almost half a century since ammonium bicarbonate was first introduced in European countries as a nitrogen fertilizer^51^. In addition, in acid soils, a marked decrease in acidity should occur in the soils which are amended with free lime to adjust the soil pH^52^. Single application of lime maintained increased soil pH and crop yield for 16 to 27 years^53^. Sun *et al.*^52^ also found ammonium bicarbonate can provide a potential strategy for ensuring healthy cucumber, watermelon and melon crops. Thus, the combined results showed that the utilization of LAB as a fumigant can effectively suppress soil nematode damage in acidic, sandy soils, especially those affected by plant-parasitic nematodes and provides a potential strategy to ensure the worldwide development of the banana industry.

## Methods

### Source of soil

Soil was collected at the “Wan Zhong” orchard (18°23′N, 108°44′E), Le Dong County, Hainan Province, China from areas monocultured with banana for more than ten years. The area has a tropical monsoon climate with approximately 600-3000 individuals of nematodes per 100 g d.w. soil. The soil was sandy and had an organic matter content of 7.6 g/kg, an available potassium content of 278.2 mg/kg, an available phosphorus content of 173.6 mg/kg, a pH value of 5.6, a total nitrogen content of 0.4 g/kg, an NH_4_^+^-N content of 10.3 mg/kg, and a NO_3_^−^-N content of 143.9 mg/kg.

### Selection of the best additive amount of ammonium bicarbonate as a fumigant to control nematodes

To select the best additive amount of the ammonia production material, ammonium bicarbonate, as a fumigant to control nematodes, eight concentrations, including 0.107 g, 0.214 g, 0.321 g, 0.428 g, 0.856 g, 1.284 g, 1.712 g and 2.568 g, were applied to pot soils (1 kg d.w. each) without banana planting and termed AB1, AB2, AB3, AB4, AB5, AB6, AB7 and AB8, respectively, were established. Because ammonium bicarbonate under alkaline conditions more readily produces volatilized ammonia, the pot soils containing ammonium bicarbonate mixed with a 2-fold concentration of lime termed LAB1, LAB2, LAB3, LAB4, LAB5, LAB6, LAB7 and LAB8, respectively, were also established. The soils in the subsequent treatments were used to compare the nematicidal effects for equivalent concentrations of ammonium bicarbonate. Pot soils with no additives, termed CK, and just containing lime, termed L1, L2, L3, L4, L5, L6, L7 and L8 were used as controls. Each treatment (16 in total) or control (9 in total) was composed of three blocks, with each block containing three pots. Each pot surface was sealed using a plastic film, leaving four ventilation holes at the bottom. The soil was mixed thoroughly with the moisture content adjusted to 20% by adding distilled water and incubated at room temperature for 15 days. After that, three subsamples combined to form a composite sample (approximately 200 g) were collected from each block for calculating the total number of nematodes.

The effects of the moisture content values (adjusted to 10%, 30%, 50% and 100%) and the temperature (adjusted to 20, 30, 40 and 50 °C) on the nematicidal ability of the mixtures of ammonium bicarbonate and lime (LAB) with the best additive concentration were examined. Pot soils without any amendment were used as controls. Treatments (8 in total) and controls (8 in total) were set as previous paragraph described. The total number of nematodes was calculated also as previous paragraph described.

### Soil chemical analysis

The pH was determined using a glass electrode meter in a suspension of 1:5 (w/v) soil: water. The total soil nitrogen content was determined by Kjeldahl digestion followed by NaOH distillation and measured by titration with 25 mM H_2_SO_4_ using boric acid as an indicator. The ammonium N (NH_4_^+^-N) and nitrate N (NO_3_^−^-N) contents were determined by extracting the soil with a 0.01 M CaCl_2_ solution (1: 10, w/v) for 30 min and then determining the NH_4_^+^-N and NO_3_^−^-N concentrations using an Auto Analyzer (Auto Analyzer 3, Germany).

### Comparison of the nematicidal effect of different ammonia production materials in Petri dishes

For the Petri dish experiment, ammonia production materials for all the treatments containing the same content of N, termed LAB (0.857 g of lime and 0.428 g of AB, d.w.), AB (0.428 g of AB, d.w.), CC (0.257 g of calcium cyanamide, d.w.) or AH (0.139 g of ammonium hydroxide, d.w.) were dissolved in 10 ml of deionized water, then 10 ml of a 20- or 50-fold dilution of each material was transferred to Petri dishes plated with 150 individual live nematodes containing the randomly nematode community with different genera collected from soil which has been planted banana more than ten years. An equal volume of deionized water was used as a control. The corrected mortality of nematodes was calculated after standing for 24 h. Nematodes were considered alive if they moved or appeared as a winding shape^54^ and were considered dead if they did not move when probed with a fine needle^55^. Then, the nematodes in each treatment were transferred to distilled water for 48 h to ascertain whether dead nematodes regained mobility or not. The corrected nematode mortality percent was calculated according to Abbott’s Formula:


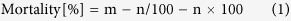


where: m and n indicate (%) mortality in treatments and the control^54^.

### Pot experiments

Pots (3 kg d.w. soil each pot) with the same content of N for LAB (0.857 g kg^−1^ soil of lime and 0.428 g kg^−1^ soil of AB, d.w.), AB (0.428 g kg^−1^ soil of AB, d.w.) and AH (0.139 g kg^−1^ soil of ammonium hydroxide, d.w.) were used as treatments and performed in a greenhouse located at Hainan Wan Zhong Co., Ltd., Hainan, China from February to May and from August to November 2013. Pot soils with or without lime (0.857 g kg^−1^ soil, d.w.), termed L and CK, respectively, were used as controls. The total number of nematodes was calculated after sealing and incubating at room temperature for 15 days. The total nematodes per 100 g d.w. soil were collected for subsequent DNA extraction prior to PCR-DGGE analysis.

After fumigating the soil, the pot soils were supplemented with cattle manure compost (30 g kg^−1^ soil, d.w.) and transplanted with banana seedlings (Musa AAA Cavendish cv. Brazil) provided by Hainan Wan Zhong Co., Ltd., China. The germ-free banana seedlings were first grown in nursery cups for 30 days and then transplanted into pots after growing 5-6 true leaves. After planting for 80 days, three plants were randomly chosen from each treatment, and the roots were uprooted from the pots and shaken gently to collect the soil samples and washed to remove the soils for the root samples. The plant parasites were collected from root. Three soil subsamples combined to form a composite sample were collected from each block, triplicate composite samples were prepared to calculate the total number of nematodes, and the total nematodes per 100 g d.w. soil were collected for subsequent DNA extraction.

### Nematode community analysis

The nematodes in the roots were extracted by using a beater to break the roots and then collected by a modified Baermann funnel methodology^56^. The nematodes in the soil were extracted using sieving and the modified Baermann funnel methodology^56^, and the nematode populations were expressed per 100 g d.w. soil. Briefly, 100 g of soil was suspended in 1.5 L of water and sieved sequentially through 0.096 mm and 0.037 mm sieves. The nematodes were collected through the 0.037 mm sieve and backwashed to 10 ml for counting using a light microscope (OLYMPUS CX41RF) × 40, the nematode taxa classified based on known feeding habitats or stoma and esophageal morphology^57^ into four functional groups: bacterivores (Ba), fungivores (Fu), plant parasites (Pp) and omnivores-predators (OP).

### DNA extraction and PCR-DGGE

The total nematodes per 100 g d.w. soil sample from the pot experiments were selected for DNA extraction by PowerSoil DNA Isolation Kits (MoBio Laboratories Inc., Carlsbad, USA) according to the manufacturer’s protocol. The three replicate DNA extractions from a combination of three replicates of soil samples collected after fumigation and at the end of the pot experiment were separately analyzed by PCR-DGGE^13^. The nematode 18S rRNA region was amplified using the primers M18F with a GC clamp (5′-AGRGGTGAAATYCGTGGAC-3′) (position 842 to 860 in relation to *Caenorhabditis elegans* 18S rRNA gene) and M18R (5′-TCTCGCTCGTTATCGGAAT-3′) (position 1,269 to 1,251 in relation to *C. elegans*)^48^. The PCR mixture and conditions were the same as those described in the original studies^49^.

DGGE analyses were conducted using a D-Code Universal Detection System (Bio-Rad Laboratories, Hercules, USA). Twenty μl of PCR product samples with 5 μl of loading dye were loaded into the wells of a 6% polyacrylamide gel (acrylamide: bis-acrylamide 37.5: 1) containing a gradient of 30% to 45% denaturants (a 100% denaturant concentration was defined as 7 M urea and 40% v/v deionized formamide). Electrophoresis was performed in 1× TAE buffer at a constant temperature of 60 °C with a constant voltage of 90 V for 10 h. The gel was visualized by silver staining and scanned using a scanner (Epson perfection v33, Seiko Epson Corporation, Japan). Strengthened intensity DGGE bands were excised and sequenced as described by Shen *et al.*^13^.

The DGGE images were analyzed by the Quantity One software program (Version 4.6.3, Bio-Rad Laboratories) for band detection and intensity measurements. Cluster analysis was performed by the UPGMA algorithm (Quantity One Version 4.6.3, Bio-Rad Laboratories).

### Data analysis

The pot experiments were repeated twice with similar results. Therefore, only the data from one of the pot experiments is presented herein. Analysis of variance (ANOVA) was used to determine differences between the different treatments with respect to nematode abundance. A comparison of means was performed by the Duncan multiple range test with a significance level of P < 0.05 using SAS9.2 (SAS Institute, Cary, N.C.).

## Additional Information

**How to cite this article**: Su, L. *et al.* Suppression on plant-parasitic nematodes using a soil fumigation strategy based on ammonium bicarbonate and its effects on the nematode community. *Sci. Rep.* **5**, 17597; doi: 10.1038/srep17597 (2015).

## Figures and Tables

**Table 1:** Chemical properties of soils from CK and from the eight treatments with different concentrations of LAB.

Treatments	pH	Total nitrogen(g/kg)	NH_4_^+^-N (mg/kg)	NO_3_^−^-N (mg/kg)
CK	5.57 ± 0.15g	0.42 ± 0.04d	9.98 ± 0.54g	143.70 ± 0.78h
1	5.87 ± 0.06f	0.36 ± 0.01e	18.54 ± 0.50c	250.99 ± 0.31g
2	6.57 ± 0.06d	0.42 ± 0.03d	28.99 ± 0.79b	348.91 ± 0.71f
3	6.33 ± 0.06e	0.46 ± 0.01c	15.35 ± 0.50de	361.74 ± 0.85e
4	6.63 ± 0.06d	0.49 ± 0.06b	14.51 ± 0.40e	486.95 ± 0.41d
5	7.03 ± 0.06c	0.47 ± 0.01c	14.48 ± 0.41e	738.01 ± 1.55c
6	7.23 ± 0.06b	0.47 ± 0.01c	11.70 ± 0.43f	856.70 ± 0.40b
7	7.26 ± 0.06b	0.49 ± 0.05b	15.69 ± 0.29d	959.77 ± 0.63a
8	8.53 ± 0.06a	0.51 ± 0.01a	136.23 ± 0.36a	29.64 ± 0.51i

Values are the means followed by standard deviation of the mean. Different letters indicate statistically significant differences at the 0.05 probability level according to the Duncan test.

**Table 2:** Nematode abundance (ind./100 g d.w. soil) of individual taxon for CK and L controls and the AB, LAB and AH treatments after fumigation and harvest (80 days after fumigation) in the pot experiment.

		After fumigation					After harvest				
		CK	L	AB	LAB	AH	CK	L	AB	LAB	AH
Bactervores	*Mesorhabditis*	13 ± 14.99b	54 ± 17.04a	40 ± 22.84ab	7 ± 3.95b	71 ± 23.95a	39 ± 23.44b	9 ± 10.40b	28 ± 12.40b	138 ± 39.49a	16 ± 16.31b
	*Acrobeloides*	111 ± 34.46a	35 ± 14.03b	46 ± 20.59b	51 ± 12.20b	25 ± 12.54b	8 ± 7.81b	30 ± 17.14b	18 ± 7.94b	172 ± 31.07a	11 ± 12.46b
	*Cephalobus*	39 ± 19.62a	7 ± 8.52b	29 ± 11.42a	7 ± 4.80b	3 ± 3.14b	44 ± 16.26b	20 ± 13.62b	39 ± 12.07b	169 ± 44.22a	11 ± 12.46b
	*Prismatolaimus*	7 ± 5.66	2 ± 3.22	4 ± 3.30	6 ± 3.27	2 ± 1.81	10 ± 11.93	14 ± 13.62	1 ± 1.98	29 ± 26.32	16 ± 8.15
	*Chiloplacus*	26 ± 11.33a	15 ± 14.03ab	2 ± 3.30b	3 ± 1.81b	2 ± 3.62b	16 ± 15.63ab	2 ± 3.93b	2 ± 3.97b	34 ± 17.23a	5 ± 4.71b
	*Acrobeles*	10 ± 9.81	15 ± 11.61	8 ± 8.72	2 ± 1.57	2 ± 1.81	5 ± 4.51	2 ± 3.93	8 ± 5.25	9 ± 8.62	14 ± 16.98
	*Monhystera*	3 ± 5.66	0 ± 0.00	0 ± 0.00	0 ± 0.00	0 ± 0.00	0 ± 0.00	0 ± 0.00	0 ± 0.00	0 ± 0.00	0 ± 0.00
	Total	209 ± 14.99a	128 ± 44.26b	128 ± 62.91b	75 ± 10.99b	106 ± 16.09b	122 ± 11.93b	77 ± 10.40b	96 ± 31.51b	551 ± 113.98a	73 ± 40.77b
Fungivores	*Tylencholaimus*	173 ± 34.46a	32 ± 17.04d	78 ± 16.49b	36 ± 7.42cd	46 ± 9.58c	86 ± 20.67a	27 ± 13.62b	16 ± 7.16b	37 ± 27.70b	5 ± 9.42b
	*Aphelenchus*	23 ± 14.99a	2 ± 3.22b	25 ± 14.37a	9 ± 4.15ab	1 ± 1.81b	0 ± 0.00	0 ± 0.00	0 ± 0.00	0 ± 0.00	0 ± 0.00
	*Aphelenchoides*	13 ± 14.99	2 ± 3.22	2 ± 3.30	0 ± 0.00	0 ± 0.00	0 ± 0.00	0 ± 0.00	0 ± 0.00	0 ± 0.00	0 ± 0.00
	Total	209 ± 29.97a	35 ± 12.88c	105 ± 17.45b	46 ± 11.03c	47 ± 11.31c	86 ± 20.67a	27 ± 13.62b	16 ± 7.16b	37 ± 27.70b	5 ± 9.42b
Plant-parasites	*Rotylenchulus*	65 ± 20.42a	37 ± 19.58ab	38 ± 14.37ab	51 ± 6.35ab	29 ± 11.01b	1529 ± 129.41a	969 ± 65.44b	936 ± 34.61b	164 ± 39.49c	1435 ± 110.32a
	*Meloidogyne*	134 ± 49.38a	152 ± 26.35a	69 ± 20.59b	53 ± 9.81b	136 ± 22.25a	471 ± 54.88a	363 ± 23.92b	60 ± 17.30d	44 ± 8.89d	266 ± 46.37c
	*Pratylenchus*	13 ± 14.99	0 ± 0.00	2 ± 3.30	0 ± 0.91	7 ± 4.79	70 ± 20.67a	9 ± 10.40b	1 ± 1.98b	0 ± 0.00b	3 ± 4.71b
	*Tylenchus*	49 ± 29.43a	7 ± 8.52c	13 ± 11.89c	7 ± 7.42c	38 ± 14.37b	0 ± 0.00	0 ± 0.00	0 ± 0.00	0 ± 0.00	0 ± 0.00
	*Ditylenchus*	0 ± 0.00	0 ± 0.00	0 ± 0.00	0 ± 0.00	0 ± 0.00	3 ± 4.51	0 ± 0.00	1 ± 1.98	0 ± 0.00	5 ± 4.71
	Total	262 ± 72.31a	197 ± 27.51ab	122 ± 34.42b	111 ± 23.62b	210 ± 45.23a	2073 ± 139.84a	1342 ± 71.10c	998 ± 45.39d	207 ± 30.09e	1710 ± 130.05b
Omnivores-Predators	*Mylonchulus*	111 ± 44.24a	74 ± 14.03ab	44 ± 20.06bc	2 ± 2.40c	8 ± 7.89c	23 ± 15.63a	9 ± 10.40ab	0 ± 0.00b	14 ± 9.95ab	0 ± 0.00b.
	*Oxydirus*	10 ± 9.81	7 ± 3.22	2 ± 3.30	0 ± 0.00	3 ± 3.14	8 ± 7.81	0 ± 0.00	2 ± 3.97	3 ± 4.97	0 ± 0.00
	*Achromadora*	0 ± 0.00	0 ± 0.00	0 ± 0.00	0 ± 0.00	0 ± 0.00	10 ± 11.93ab	20 ± 13.62a	5 ± 1.98b	3 ± 4.97b	0 ± 0.00b
	*Aporcelaimus*	23 ± 14.99a	4 ± 6.44b	11 ± 5.71ab	4 ± 3.27b	4 ± 4.79b	0 ± 0.00	0 ± 0.00	0 ± 0.00	0 ± 0.00	0 ± 0.00
	*Microdorylaimus*	0 ± 0.00	0 ± 0.00	0 ± 0.00	0 ± 0.00	0 ± 0.00	3 ± 9.02b	7 ± 25.33b	0 ± 0.00b	29 ± 18.97a	0 ± 0.00b
	Total	144 ± 49.38a	86 ± 19.58b	57 ± 15.11bc	6 ± 5.66d	16 ± 10.86cd	44 ± 9.02a	36 ± 20.80a	7 ± 5.95b	49 ± 13.16a	0 ± 0.00b
Total nematodes		824 ± 13.91a	446 ± 18.11b	411 ± 19.78c	237 ± 12.51d	378 ± 27.47c	2326 ± 17.44a	1482 ± 30.79c	1117 ± 33.03d	945 ± 14.79e	1789 ± 45.59b

Values are the means followed by standard deviation of the mean. Different letters indicate statistically significant differences at the 0.05 probability level according to the Duncan test.

CK, pot soils with no additives; L, lime; AB, ammonium bicarbonate; LAB, combination of lime and ammonium bicarbonate; AH, ammonium hydroxide.

**Table 3:** Closest relative for the DGGE bands with the greatest similarities in BLAST database search after fumigation from the pot experiment.

	Band number	Accession	Species	Similarity (%)
Bacterial-feeders	N1	EU040133.1	*Prismatolaimus intermedius*	98
	N2	AF202161.1	*Cephalobus cubaensis*	98
	N3	JQ237850.1	*Acrobeloides maximus* strain RT2-R25A	92
	N4	EU306344.1	*Acrobeloides maximus* isolate wb4	92
	N6	U81576.1	*Acrobeles*	99
	N9	AY284662.1	*Cephalobus persegnis* isolate CephPer1	97
	N10	AY284671.1	*Acrobeles complexus* isolate AcroCom1	99
	N13	JQ957905.1	*Acrobeloides* cf. buetschlii 1 JH-2012	96
Plant-parasites	N5	EU306348.1	*Tylenchus arcuatus* isolate wb8	91
	N7	AY284635.1	*Ditylenchus brevicauda* isolate DityBre	91
	N8	KP054074.1	*Rotylenchulus reniformis* isolate ZJ7 clone 7	98
	N11	AJ966486.1	*Helicotylenchus dihystera*	99
	N12	JX406362.1	*Rotylenchulus reniformis* isolate 25A clone 10 pop-variant RN_VAR1	97
	N14	AF442193.1	*Meloidogyne javanica*	95
	N15	U81578.1	*Meloidogyne incognita*	96

**Table 4:** Closest relative for the DGGE bands with the greatest similarities in BLAST database search at the end of the pot experiment.

	Band number	Accession	Species	Similarity (%)
Bacterial-feeders	N1	JQ237850.1	*Acrobeloides maximus* strain RT2-R25A	92
	N2	EU306344.1	*Acrobeloides maximus* isolate wb4	92
	N5	AY284671.1	*Acrobeles complexus* isolate AcroCom1	99
	N9	AY284662.1	*Cephalobus persegnis* isolate CephPer1	97
	N10	AY284671.1	*Acrobeles complexus* isolate AcroCom1	99
	N13	FJ716584.1	*Mesorhabditis* sp. WB-2009	99
Plant-parasites	N3	EU306348.1	*Tylenchus arcuatus* isolate wb8	91
	N4	EU368587.1	*Tylenchorhynchus claytoni*	91
	N6	AY284635.1	*Ditylenchus brevicauda* isolate DityBre	91
	N7	KC618471.1	*Heterodera elachista*	91
	N8	JX406362.1	*Rotylenchulus reniformis* isolate 25A clone 10 pop-variant RN_VAR1	98
	N11	JX406383.1	*Rotylenchulus reniformis* isolate 13B clone 20 pop-variant RN_VAR2	99
	N12	AY284606.1	*Helicotylenchus pseudorobustus* isolate HeliPse	96
	N14	AF442193.1	*Meloidogyne javanica*	95
	N15	U81578.1	*Meloidogyne incognita*	96
